# Hinge Craniotomy for Posterior Cranial Vault Expansion: Using the Keel to the Surgeon’s Advantage

**DOI:** 10.7759/cureus.64110

**Published:** 2024-07-08

**Authors:** William Coggins, Sagar Mehta, Tomoko Tanaka

**Affiliations:** 1 Neurosurgery, University of Arkansas for Medical Sciences, Little Rock, USA; 2 Plastic and Reconstructive Surgery, University of Arkansas for Medical Sciences, Little Rock, USA

**Keywords:** occipital keel, occipital bone, hinge craniotomy, craniofacial, cranial vault reconstruction

## Abstract

Cranial vault reconstructions are a common craniofacial procedure utilized to treat chronically elevated intracranial pressure and its sequelae for children with craniosynostosis. These surgeries often involve split-thickness autologous grafts to facilitate intracranial volume expansion. The hinge craniotomy was developed by neurosurgeons in the early 2000s as an alternative to the hemicraniectomy to allow for greater space and simplified re-securing of the bone flap. In our report, we introduce a novel application of hinge craniotomy in total cranial vault reconstruction for a pediatric patient with microcephaly secondary to congenital cytomegalovirus infection. We performed bilateral barrel stave cuts to the occipital bone as well as an undercut along the midline keel to form a hinge craniotomy. Complex reconstruction followed to augment intracranial volume and restructure the cranial vault. This technique maximized intracranial volume expansion while minimizing the need for prolonged reconstruction. It also allowed for retained vascularization of the bone flap by maintaining the connection with the intact cranial base and pericranium to further support bony healing. Our study presents a novel utilization of hinge craniotomy, using the occipital keel as a natural hinge, to create ample space during cranial vault reconstruction. This technique offers potential advantages in terms of intracranial volume expansion and bony healing.

## Introduction

Complex cranial vault reconstructions require extensive planning and attention to detail during intra-operative reconstruction. These cases are ideally planned and performed by a multi-disciplinary craniofacial team including neurosurgeons and plastic/reconstructive surgeons. The overarching goal of cranial vault reconstruction is to expand the intracranial volume to provide relief of chronically elevated intracranial pressure [1]. Split-thickness autologous bone grafts are often utilized to create large increases in the intracranial volume. However, these bone grafts are completely removed from the native cranium resulting in several disadvantages including complete vascular disconnection, greater risk of infection, and the requirement of splitting the bone into two cortical halves via the spongy cancellous bone [2,3].

Hinge craniotomy first appeared in the neurosurgical literature in the early 2000s as an alternative to the standard hemicraniectomy oftentimes performed for trauma or post-stroke [4,5] Multiple techniques have been described including suturing the flap loosely in place, drilling off the inner table to provide more room, and intentionally bending plates to elevate the bone flap [6-9]. The most common description entailed replacing the bone flap inferiorly with plates and screws but leaving the superior edge disconnected to allow for progressive edema. As expected, these advances in technique conferred several advantages including no longer needing to store a bone flap and simpler cranioplasties. This historical technique provides a groundwork for our modification and adaptation of it into cranial vault reconstructions. The concept of hinging a bone flap is foundational to the surgery described in which we preserve vascular and bony integrity to the cranial base to enhance healing. In this report, we describe a novel technique by creating a hinge craniotomy centered on the midline keel during a total cranial vault reconstruction.

## Case presentation

Our patient was a three-year-old female who suffered from congenital cytomegalovirus, resulting in cerebral palsy and subsequent premature suture closure, leading to microcephaly. The patient developed behavioral changes such as head banging, irritability, and inconsolable crying. Due to significant developmental delay (speech delay and hearing impairment), expression of symptoms was limited. However, after a thorough exploration of alternative causes, we determined that her symptoms were manifestations of increased intracranial pressure.

**Figure 1:**
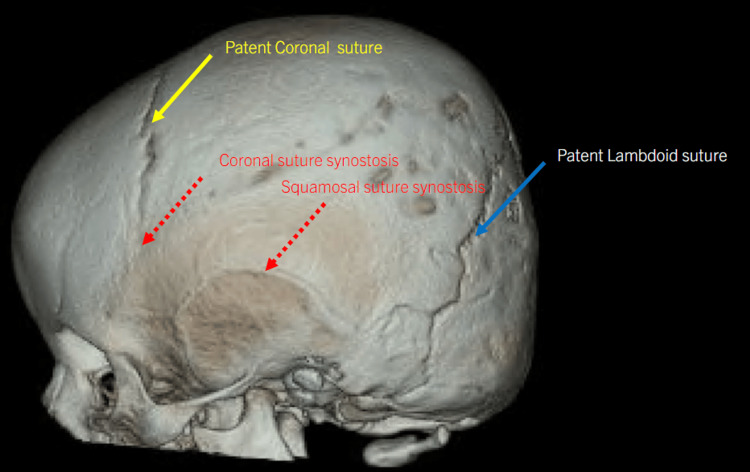
CT scan and three-dimensional reconstruction of the skull demonstrating microcephaly and craniosynostosis of the metopic, squamosal, and bicoronal (partial) sutures and patent lambdoid suture.

**Figure 2:**
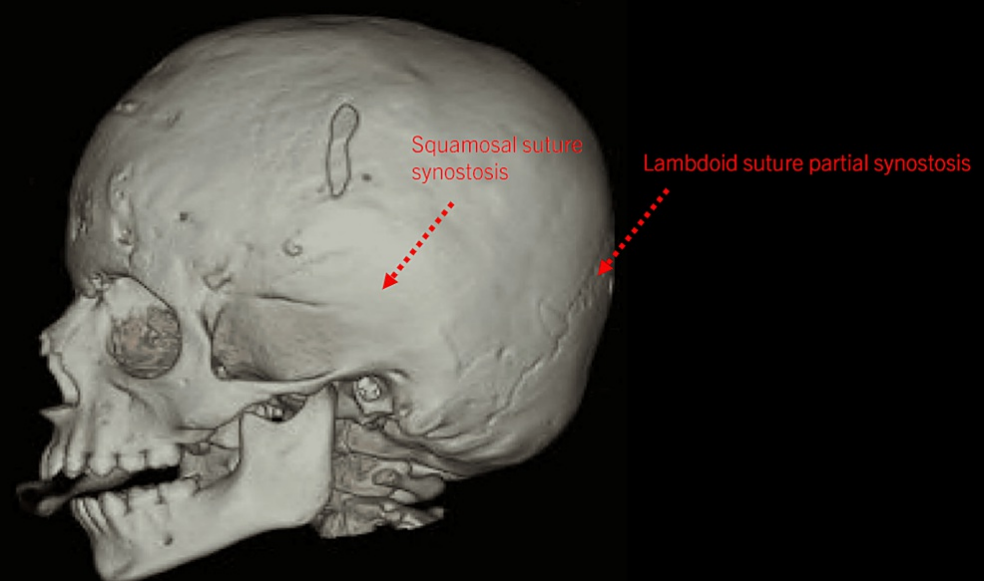
CT scan and three-dimensional reconstruction of the skull post anterior cranial vault remodeling with good expansion of anterior vault but with subsequent lambdoid synostosis and flattening of occiput.

**Figure 3:**
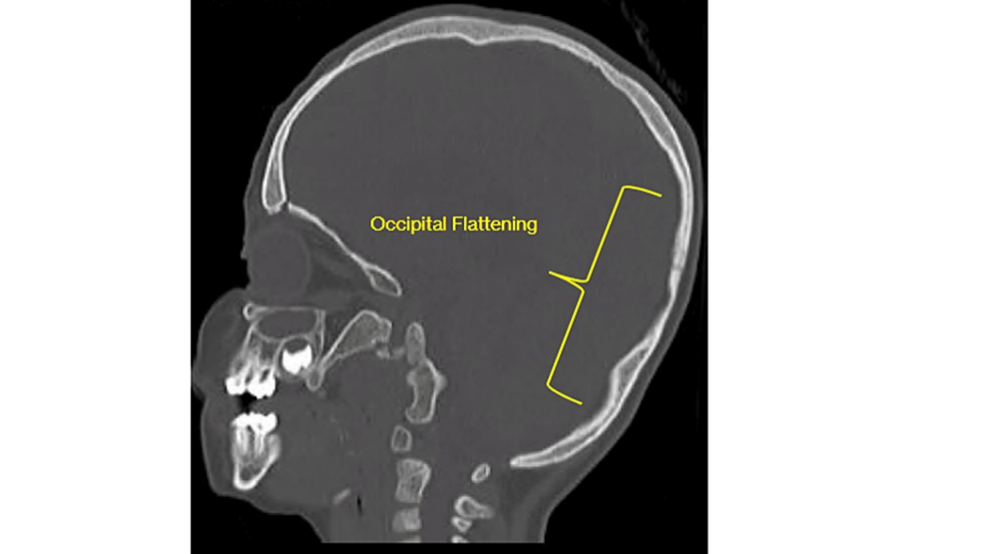
Head CT sagittal view on bone windows demonstrating occipital flattening.

The patient previously underwent anterior cranial vault remodeling with frontal-orbital bar advancement to address metopic, squamosal, and partial bicoronal synostosis, at 12 months of age by another neurosurgeon as evidenced in Figure 1. Unfortunately, there was subsequent development of partial lambdoid synostosis and occipital flattening (Figures 2-3).

A planned posterior cranial vault expansion was thus arranged, roughly two years after the index surgery, when the child was three years old. The patient presented to the operating theater for planned posterior cranial vault expansion.

Surgical technique

**Figure 4:**
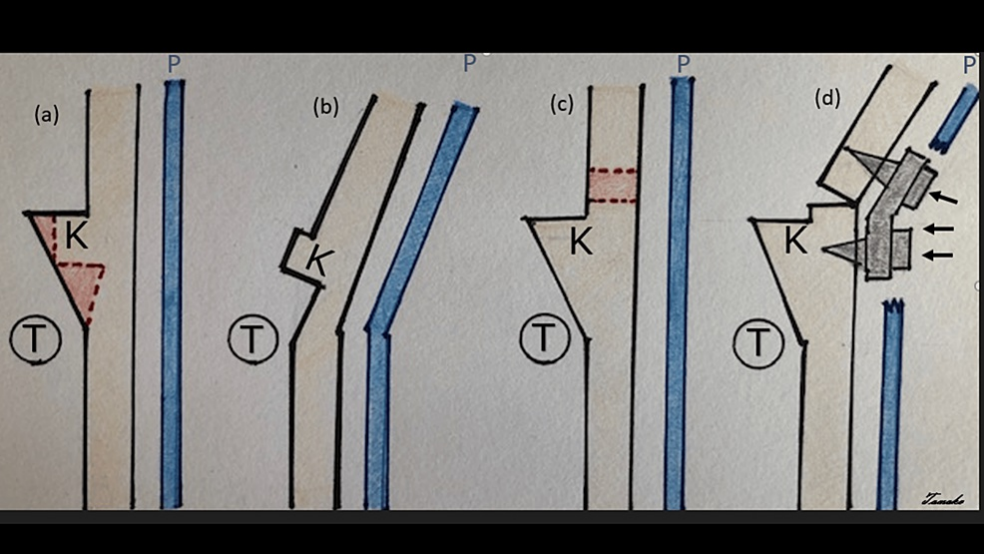
Schematic view of surgical techniques (a) Present technique of planning undercut (red dots line) of the occipital keel. (b) After undercutting the occipital keel and smoothed edge, a hinge opens the occipital bone with intact periosteum. (c) Conventional technique of planning craniotomy cut (red dots line)  above the Torcula. (d) Fixation by plate and screws (small black arrows) for craniotomy reconstruction. The pericranium was disrupted due to craniotomy. K: occipital keel; T: torcula; P: pericranium Image Credits: Tomoko Tanaka, MD

**Figure 5:**
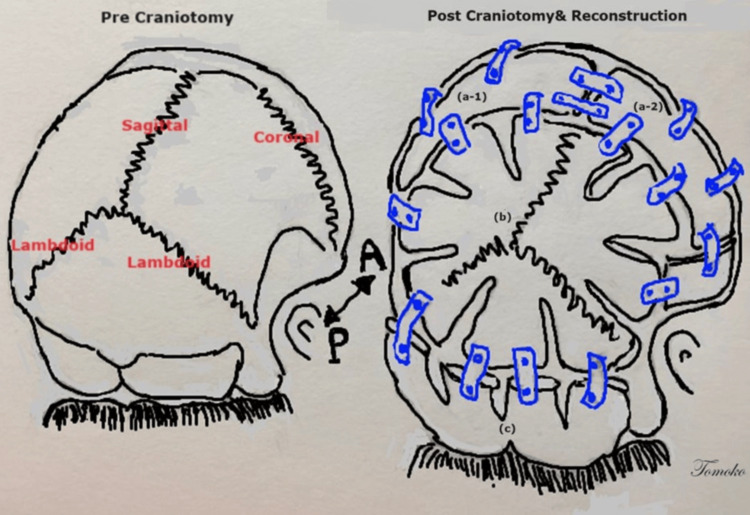
Schematic view before the second craniotomy and after the craniotomy and reconstruction Pre-craniotomy on left with coronal, sagittal, lambdoid suture. Post-craniotomy and reconstruction on right. (a-1) and (a-2) sections were created by split thickness grafts. (b) Posterior parietal and occipital bone were expanded by barrel-stave osteotomies. (c) Hinge craniotomy section with undercutting keel and barrel-stave osteotomies to separate into four parts. Reconstruction was proceeded by utilize disposable flexible plate and screws. A: anterior; P: posterior Image Credits: Tomoko Tanaka, MD

After intubation and appropriate line placement by the anesthesia team, the patient was placed in the sphinx position. The previous incision was utilized; a pericranial flap was preserved for closure. The posterior cranial vault was sequentially opened in four separate pieces. Bone flaps were then split using a Misonix BoneScalpel® (Innosurge, Randers, Denmark) to produce split-thickness grafts. These grafts were then set aside for use during the reconstructive portion of the surgery. The underlying dura in bilateral occipital regions over the transverse sinus and torcula was carefully dissected free with a Penfield dissector #1. The craniotome drill was then employed to create barrel stave cuts bilaterally directed in the axial plane horizontally toward the midline. These cuts were made in the shape of an “L” with the long arm directed to the midline. The midline occipital keel was then elevated with a partial undercut in the inner cortical table along the keel. The section was reflected outward with a Tessier bone bender to create a greenstick fracture in the occipital bone and ultimately, a hinge craniotomy (Figure 4). This maneuver greatly expanded the occipital region while maintaining an intact connection to the cranial base. A complex reconstruction was then performed attaching the split-thickness grafts with resorbable plates and screws (Stryker Corporation, Kalamazoo, Michigan, United States) anteriorly to the vault and posteriorly to the hinge craniotomy. Our reconstruction augmented the intracranial volume and nicely refashioned the cranial vault (Figure 5).

The section was reflected outward with a Tessier bone bender to create a greenstick fracture and ultimately, a hinge craniotomy. This maneuver greatly expanded the occipital region while maintaining an intact connection to the cranial base. A complex reconstruction was then performed to augment the intracranial volume and restructure the cranial vault. 

Postoperatively, the patient had a pressure headwrap in place to minimize postoperative edema for several days. At the one-month follow-up with the craniofacial team, the patient was recovering well from surgery, and at the 1.5-year (approximately) follow-up with the rehabilitation service, she was progressing slowly with her development and motor skills including standing with support and cruising. 

## Discussion

Optimizing cranial vault reconstructions requires carefully executing select osteotomies to maximize intracranial volume expansion while maintaining bony integrity. Timeliness is paramount in these long surgeries to minimize blood loss, decrease transfusion requirements, and circumvent intensive care unit admissions postoperatively. With our technique, we maximized intracranial volume expansion without resorting to additional split-thickness grafts. Instead, the hinge craniotomy in combination with parietal split-thickness grafts created maximal space in an expeditious fashion while protecting the torcula. Our technique does not incur any additional cost as we used the same surgical instruments to perform it. In fact, it could diminish cost by requiring fewer cranial plates and screws given the maintenance of a connection to the cranial base. Similarly, it does not require any new surgical skills to perform, just an understanding and exploitation of the anatomy of the occipital bone. We expect our technique to pos no additional increased risk for postoperative infection or iatrogenic damage to ventriculoperitoneal shunt hardware, if in place. This series of cuts could be useful in cases of syndromic or non-syndromic craniosynostosis.

We performed a total of 54 cranial vault remodeling procedures in 2022. Of those, 32 had recorded procedure times. On average, a cranial vault expansion at our institution in 2022 took 264 minutes. In the case presented, the procedure time was 292 minutes. While this was a longer operative time than our average, it was the second cranial operation for our patient, and navigating scarring in the scalp and dura explains the added operative time. An additional benefit to the hinge method obviates the need for bony cuts adjacent to the torcula, a potential risk for sinus injury. Because the occipital keel is usually thick and inflexible to molding, undercutting it and creating a hinge to out-fracture barrel stave cuts allows for the mobility to expand the vault posteriorly without completely removing the large scaffold of squamous occipital bone necessary to create split-thickness grafts.

Another advantage of our technique resides in bony healing. Bone flap resorption rates increase significantly with age younger than two and a half years [10]. For the pediatric population, resorption of the bone flap creates an additional burden on families, and the choices of allograft synthetic materials are limited. Surgical means to mitigate resorption have yet to be developed [11,12]. Bone healing occurs primarily through three means: direct osteogenesis, osteoconduction, and osteoinduction. In our practice, we preserve the pericranial layer and close it as a flap over the reconstruction to augment osteoinduction and osteogenesis. Studies on fracture healing demonstrate that indirect bony healing occurs through an endochondral scaffold [13]. This framework is formed from mesenchymal stem cell migration to the immediate hematoma present post injury. It subsequently undergoes ossification and ultimately, lamellar remodeling. We hope by maintaining the close anatomical relationship of the pericranium to the newly created cranial vault, bone healing is augmented. Prior craniofacial literature also demonstrates that bone resorption and other complications are typically related to non-vascularization of the graft or implant [14,15]. In our technique, we employ a greenstick fracture to create a hinge craniotomy. We expect maintaining the connection with the intact cranial base will augment bony healing via retained vascularization.

## Conclusions

We report a novel utilization of the hinge craniotomy to provide a wide degree of space in the occipital region. This technique was made possible by exploiting the occipital keel to function as a natural hinge. By maintaining an intact osseous and vascular connection to the cranial base, we expect our technique will augment bony healing. In conjunction with the anatomic healing advantages, we expect exploitation of the occipital keel to diminish operative times in posterior cranial vault reconstruction by creating major volume expansion with a series of quick cuts rather than additional time-consuming split-thickness grafts. Of course, future comparative studies with traditional osteotomies are necessary to confirm superiority. We hope to add a simple yet effective tactic to the operative arsenal of craniofacial teams by reporting this novel technique.
